# Glutamic acid decarboxylase autoantibodies are dominant but insufficient to identify most Chinese with adult-onset non-insulin requiring autoimmune diabetes: LADA China study 5

**DOI:** 10.1007/s00592-015-0799-8

**Published:** 2015-08-05

**Authors:** Yufei Xiang, Gan Huang, Zhongyan Shan, Linlin Pan, Shuoming Luo, Liyong Yang, Lixin Shi, Qifu Li, R. David Leslie, Zhiguang Zhou

**Affiliations:** Institute of Metabolism and Endocrinology, The Second Xiangya Hospital, Key Laboratory of Diabetes Immunology, Ministry of Education, Central South University, National Clinical Research Center for Metabolic Diseases, Changsha, 410011 China; Department of Endocrinology and Metabolism, Institute of Endocrinology, Liaoning Provincial Key Laboratory of Endocrine Diseases, The First Affiliated Hospital of China Medical University, Shenyang, China; The Endocrinology Department, The First Affiliated Hospital of Fujian Medical University, Fuzhou, China; Section of Endocrinology, Affiliated Hospital of Guiyang Medical College, Guiyang, China; Department of Endocrinology, The First Affiliated Hospital of Chongqing Medical University, Chongqing, China; Department of Diabetes and Metabolic Medicine, Blizard Institute, London, UK

**Keywords:** Latent autoimmune diabetes of adults (LADA), Glutamic acid decarboxylase antibody (GADA), Protein tyrosine phosphatase-2 antibody (IA-2A), Insulin autoantibody (IAA), Zinc transporter 8 autoantibody (ZnT8A)

## Abstract

**Aims:**

Adult-onset autoimmune diabetes is prevalent in China, in contrast to childhood-onset type 1 diabetes mellitus. Islet autoantibodies are the most important immune biomarkers to diagnose autoimmune diabetes. We assayed four different islet autoantibodies in recently diagnosed adult non-insulin-requiring diabetes Chinese subjects to investigate the best antibody assay strategy for the correct diagnosis of these subjects.

**Methods:**

LADA China study is a nation-wide multicenter study conducted in diabetes patients from 46 university-affiliated hospitals in China. Non-insulin-treated newly diagnosed adult diabetes patients (*n* = 2388) were centrally assayed for glutamic acid decarboxylase autoantibody (GADA), protein tyrosine phosphatase-2 autoantibody (IA-2A), and zinc transporter 8 autoantibody (ZnT8A) by radioligand assay and insulin autoantibody (IAA) by microtiter plate radioimmunoassay. Clinical data were determined locally.

**Results:**

Two hundred and six (8.63 %) subjects were autoantibody positive, of which GADA identified 5.78 % (138/2388) of the total, but only 67 % (138/206) of the autoimmune cases. IA-2A, ZnT8A, and IAA were found in 1.51, 1.84, and 1.26 % of the total study subjects, respectively. When assaying three islet autoantibodies, the most effective strategy was the combination of GADA, ZnT8A, and IAA, which could identify 92.2 % (190/206) autoimmune diabetes patients. The clinical data showed that those subjects with positive GADA had lower random C-peptide than autoantibody negative subjects (*P* < 0.05).

**Conclusions:**

As with Europeans, GADA is the dominant autoantibody in this form of autoimmune diabetes in China, but in contrast to Europeans, screening should include other diabetes-associated autoantibodies.

## Introduction

Adult-onset autoimmune diabetes, associated with diabetes-associated autoantibodies, often presents a similar clinical phenotype to type 2 diabetes mellitus [1–3]. In patients with this clinical phenotype, known as latent autoimmune diabetes of adults (LADA), β cell function decreases about threefold faster than in type 2 diabetes [4]. As a result, treatment needs to be implemented early and aggressively, yet even on insulin therapy such patients can show worse diabetes control [5–7]. Early therapeutic intervention requires early diagnosis. For LADA, the diagnostic criteria are controversial [8], but the widely accepted criteria from the Immunology of Diabetes Society (IDS) are: (1) adult-onset (>30 years), (2) insulin independency at diagnosis, and (3) positive islet autoantibodies [2, 9].

Although the genetic background contributed a lot to the onset of autoimmune diabetes, islet autoantibodies are the most widely used in practice for the diagnosis [10]. Of the most widely studied diabetes-associated autoantibodies in LADA, islet cell antibody (ICA) [11] and glutamic acid decarboxylase antibody (GADA) are the most prevalent in Europeans [12, 13], though a proportion (about 6 %) only have protein tyrosine phosphatase-2 antibody (IA-2A), insulin autoantibody (IAA) [14, 15], or zinc transporter 8 autoantibody (ZnT8A) [16–18]. We found that adult-onset autoimmune diabetes is prevalent in China when testing for GADA [1], while ZnT8A augments the detection of LADA in such Chinese patients [17]. We have noted differences in the relative frequency of diabetes-associated autoantibodies in European and Chinese childhood-onset type 1 diabetes mellitus patients [19]. To determine the most appropriate islet autoantibody assaying strategy to identify Chinese patients with LADA from adult-onset non-insulin requiring diabetes patients, we assayed a large cohort for four different diabetes-associated autoantibodies.

## Research design and methods

LADA China is the first multicenter study to investigate adult-onset autoimmune diabetes in recently diagnosed phenotypic type 2 diabetes mellitus subjects in China in terms of the epidemiology, clinical features, and immunogenetic characteristics [1]. From this patient cohort (*n* = 4880), we assayed 2388 non-insulin-requiring recently diagnosed diabetes patients with sufficient serum. The inclusion criteria included: (a) 1999 WHO diabetes diagnostic criteria; (b) disease-onset not less than 30 years old; (c) ascertained within 1 year from diagnosis; (d) without ketoacidosis within 6 months after diagnosis; (e) not on insulin therapy after diagnosis. The exclusion criteria included: (a) not with secondary diabetes; (b) women not in pregnancy; (c) not with tumor; (d) not with severe heart, renal, or liver dysfunction. Demographics are shown in Table 1; clinical and laboratory data including age, gender, height, weight, waist circumferences, hip circumferences, HbA1c, cholesterol (CHOL),triglyceride (TG), LDL-chol, HDL-chol, and fasting C-peptide (FCP) were collected locally with standardized methods by trained physicians and clinical biochemistry laboratories.

**Table 1:** Clinical characteristics of antibody positive LADA and antibody negative type 2 diabetes mellitus subjects

	Ab (−) type 2 diabetes mellitus	Ab (+) LADA
*n*	2182	206
Gender (M/F)	1250/932	129/77
Age (years)	51.2 ± 11.0	50.8 ± 11.6
BMI (kg/m^2^)	25.1 ± 3.8	24.6 ± 3.5
WHR	0.91 ± 0.07	0.91 ± 0.07
TG (mmol/L)	1.82 (0.39–34.13)	1.68 (0.50–14.99)^a^
CHOL (mmol/L)	5.05 ± 1.19	5.18 ± 1.18
HDL-C (mmol/L)	1. 27 ± 0.55	1.39 ± 0.74
LDL-C (mmol/L)	2.89 ± 0.94	2.89 ± 0.97
SBP (mmHg)	127.6 ± 18.1	125.6 ± 18.4
DBP (mmHg)	81.0 ± 18.5	80.2 ± 11.1
HbA1c (%)	8.77 ± 2.87	9.00 ± 2.52
Converted mean HbA1c (mmol/mol)	72	75
FCP (pmol/L)	657.3 (5.4–3210.4)	573.7 (59.44–1949.59)

^a^Compared with Ab (−) group. *P* < 0.05

All study subjects will centrally assayed for GADA, IA-2A, ZnT8A, and IAA using standardized assays in a core laboratory (Diabetes Center, Central South University). The GADA, IA-2A, and ZnT8A were measured by radioligand assay (RLA), and IAA was by microtiter plate radioimmunoassay (RIA). In the Diabetes Autoantibody Standardized Programme (DASP) 2009, all our assays showed high sensitivity and specificity, i.e., GADA assay (sensitivity 72 %, specificity 98 %), IA-2A assay (sensitivity 66 %, specificity 99 %), ZnT8A assay (sensitivity 66 %, specificity 100 %), and IAA assay (sensitivity 42 %, specificity 98 %), respectively.

## Statistics

All analyses were performed by SPSS 13.0 software. Results were shown as mean ± SD or as median (range) or otherwise documented as positive cases, constituent ratio or ratio. The unpaired *t* tests and analysis of variance for multiple comparisons were used for normally distributed data, and nonparametric tests were used for non-normally distributed data. The difference between classified variables was tested using Chi-squared test or Fisher’s exact test if the expected number of subjects in any cell was less than 5. *P* values less than 0.05 were considered significant.

## Results

### Frequency of four islet autoantibodies in non-insulin requiring adult-onset diabetes

Overall, in non-insulin requiring diabetes patients: positivity of GADA, IA-2A, ZnT8A and IAA were 5.8, 1.5, 1.8, and 1.3 % respectively. We stratified all these patients by age, i.e., 30–39, 40–49, 50–59 and over 60 years; the four islet autoantibodies did not show any statistical differences between these age subgroups. However, in every age subgroup, positivity for GADA is higher than for the other three autoantibodies (*P* = or < 0.01 for each) (Table 2).

**Table 2:** Age stratification of the non-insulin-requiring diabetes patients with the positivity for GADA, IA-2A, ZnT8A, and IAA

Age (years)	Cases	GADA	IA-2A	ZnT8A	IAA
30–39	373	6.17 % (23/373)	1.34 % (5/373)^a^	2.14 % (8/373)^a^	1.07 % (4/373)^b^
40–49	718	5.85 % (42/718)	2.09 % (15/718)^b^	2.51 % (18/718)^a^	1.11 % (8/718)^b^
50–59	753	5.58 % (42/753)	1.06 % (8/753)^b^	1.33 % (10/753)^b^	1.60 % (12/753)^b^
≥60	544	5.70 % (31/544)	1.47 % (8/544)^b^	1.47 % (8/544)^b^	1.10 % (6/544)^b^
In total	2388	5.78 % (138/2388)	1.51 % (36/2388)^b^	1.84 % (44/2388)^b^	1.26 % (30/2388)^b^

Data were showed as percentage (positive cases/total cases), compared with the GADA positivity. ^a^ *P* < 0.01, ^b^ *P* < 0.001

### Association of four islet autoantibodies in recently diagnosed adult-onset non-insulin-requiring diabetes

**Fig. 1:**
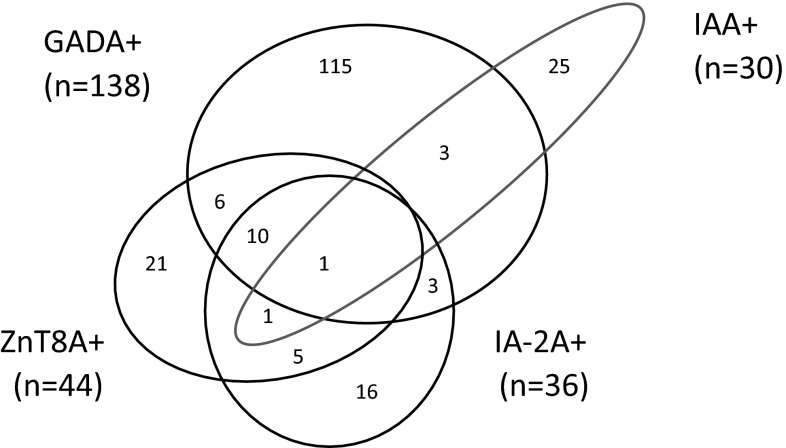
Venn diagram of GADA, IA-2A, ZnT8A, and IAA positivity in adult non-insulin requiring autoimmune diabetes patients

**Fig. 2:**
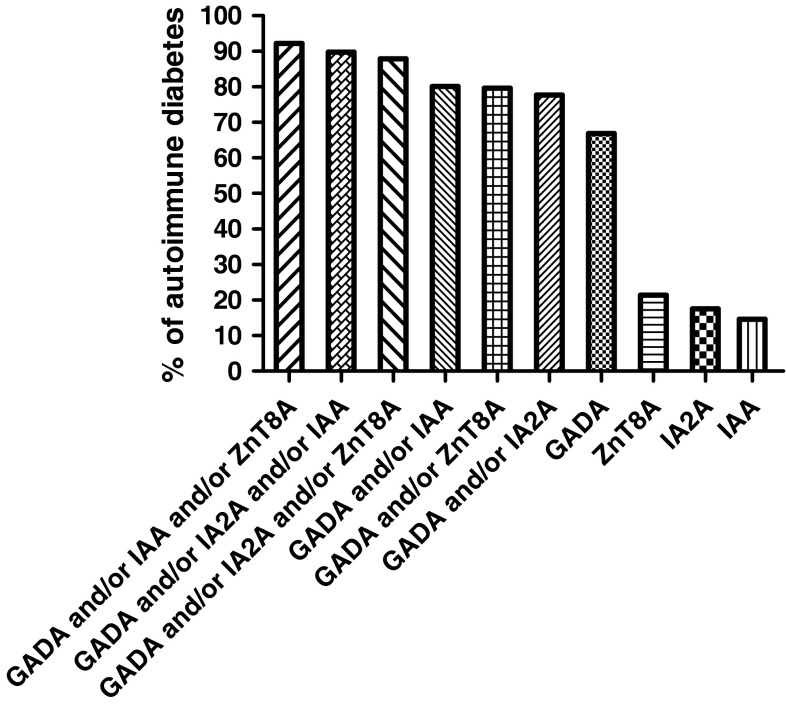
Histogram of different assay strategy for GADA, ZnT8A, IA-2A, and IAA in adult-onset autoimmune diabetes. *x* axis stands for different diabetes-associated islet autoantibodies combination strategies. *y* axis stands for the percentage of patients with different assay strategies in a total of 206 adult autoimmune diabetes

Of 2388 patients studied, 206 cases (8.6 %) had islet autoantibodies, of which the majority (177/206; 85.9 %) had a single positive autoantibody, more than had two autoantibodies (17/206; 8.3 %), in turn, more than had three autoantibodies (11/206; 5.3 %). Only one case had all four islet autoantibodies. Overlap of positive autoantibodies is shown in Fig. 1. Of note the majority of autoantibody positive cases had GADA (138/206, 67 %) (Fig. 2). Of 206 patients with autoantibodies, a combination assay with GADA plus one other autoantibody could identify 80.1 % (with IAA), 79.6 % (with ZnT8A), and 77.7 % (with IA2A), respectively (Fig. 2).The hierarchy of likelihood for detecting autoimmune diabetes by autoantibody testing in this cohort was GADA > IAA > ZnT8A > IA2A. When assaying this cohort with three islet autoantibodies, the most effective strategy was the combination of GADA, ZnT8A, and IAA, which could identify about 92.2 % (190/206) of autoimmune diabetes patients of the total of 8.6 % with autoantibodies (Fig. 2). We have investigated the positivity of IAA, ZnT8A, and IA2A among high and low GADA titer subgroups, and there was no statistical differences for the presence of these autoantibodies in the different GADA titer groups (data not shown).

### Clinical features of subjects with autoantibodies

Compared with type 2 diabetes mellitus patients, clinical and laboratory characteristics of adult-onset autoimmune patients were similar, apart from having lower triglycerides (Table 1). Patients with GADA had lower β cell function as estimated by fasting C-peptide (FCP) [controls vs. GADA (+): 657.3 (5.4–3210.4) vs. 519.3 (105.9–1648.0) pmol/L, *P* < 0.001], higher HDL-ch [controls vs. GADA (+):1.27 ± 0.55 vs. 1.42 ± 0.82 mmol/L, *P* < 0.05], and lower SBP [controls vs. GADA (+): 127.6 ± 18.1 vs. 123.0 ± 17.2 mmHg, *P* < 0.05] (Table 3). Though the numbers were small, patients with adult autoimmune diabetes with single autoantibodies, when compared with type 2 diabetes mellitus patients, had significantly lower BMI (with IA2A alone), lower triglycerides (with IA2A or IAA alone), higher HDL (with IA2A alone), higher CHOL (with ZnT8A alone), and higher WHR (with ZnT8A alone)(*P* < 0.05 for each) (Table 3). Compared with GADA (+) autoimmune diabetes subjects, patients with ZnT8A had higher WHR and SBP (*P* < 0.05 for each) (Table 3).

**Table 3:** Clinical characteristics of GADA, IA-2A, ZnT8A, and IAA positive and autoantibody negative non-insulin required type 2 diabetes mellitus subjects

	Abs (−)	GADA (+)	IA-2A (+) GADA (−)	ZnT8A (+) GADA (−)	IAA (+) GADA (−)
*n*	2182	138	22	27	26
Gender (M/F)	1250/932	84/54	12/10	18/9	19/7
Age (years)	51.2 ± 11.0	50.48 ± 11.6	52.1 ± 12.3	49.56 ± 10.06	53.6 ± 12.7
BMI (kg/m^2^)	25.1 ± 3.8	24.7 ± 3.8	23.6 ± 2.5^b^	24.8 ± 3.1	24.6 ± 2.7
WHR	0.91 ± 0.07	0.90 ± 0.07	0.92 ± 0.06	0.94 ± 0.04ae	0.90 ± 0.06
TG (mmol/L)	1.82 (0.39–34.13)	1.70 (0.50–14.99)	1.54 (0.67–2.49)^cd^	1.73 (0.88–13.36)	1.64 (0.64–4.74)^a^
CHOL (mmol/L)	5.05 ± 1.19	5.19 ± 1.22	5.17 ± 1.07	5.56 ± 1.13^a^	4.87 ± 1.07
HDL-C (mmol/L)	1.27 ± 0.55	1.42 ± 0.82^a^	1.50 ± 0.42^a^	1.37 ± 0.68	1.28 ± 0.45
LDL-C (mmol/L)	2.89 ± 0.94	2.84 ± 1.01	2.99 ± 0.72	3.25 ± 1.02	2.82 ± 0.76
SBP (mmHg)	127.6 ± 18.1	123.0 ± 17.2^a^	124.0 ± 14.9	133.7 ± 23.4e	131.9 ± 16.3
DBP (mmHg)	81.0 ± 18.5	79.55 ± 11.0	80.3 ± 8.7	83.6 ± 13.0	80.2 ± 10.5
HbA1c (%)	8.77 ± 2.87	9.04 ± 2.39	10.18 ± 2.51	9.20 ± 2.16	7.48 ± 3.09
Converted Mean HbA1c (mmol/mol)	72	75	88	77	58
FCP (pmol/L)	657.3 (5.4–3210.4)	519.3 (105.9–1648.0)^b^	685.2 (427.0–897.0)	887.1 (197.0–1949.6)	626.0 (59.44–1774.16)

Compared with Abs (−) group. ^a^ *P* < 0.05, ^b^ *P* < 0.01, ^c^ *P* < 0.001; compared with GADA (+) group, ^d^ *P* < 0.05, ^e^ *P* < 0.01

## Conclusions

Our previous studies in childhood-onset type 1 diabetes mellitus suggested that the pattern of islet autoantibodies in Chinese patients is different from European patients [18]. In European childhood-onset type 1 diabetes mellitus subjects, diabetes-associated autoantibodies are prevalent, e.g., GADA (79 %), IA-2A (69 %), ZnT8A (64 %), and IAA (70 %) [20]. In contrast, in Chinese patients the same autoantibodies are less prevalent: GADA (53 %), IA-2A (26 %), ZnT8A (24 %), and IAA (22 %) [18]. In both populations, GADA was the most prevalent autoantibody. However, it is well established that the pattern of autoantibodies differs in European adult-onset, compared with childhood-onset, autoimmune diabetes [18]. In particular, IAA is prevalent in children but far less prevalent in adults, while GADA is by far the most prevalent autoantibody in adult-onset autoimmune diabetes [1, 21, 22]. Since such autoimmune diabetes patients are easily misdiagnosed as having type 2 diabetes mellitus, it is important to determine the optimum screening strategy for them. This present report is the first to describe the pattern of multiple diabetes-associated autoantibodies in non-insulin-requiring adult-onset diabetes in a Chinese population.

In the European Action LADA study, 90 % of adult-onset diabetes patients had GADA, while IA-2A and ZnT8A only identified 10 % of the remaining cases [3]. In the Italian NIRAD study, 96 % of adult-onset autoimmune diabetes patients had GADA, the remainder being identified by IA-2A and ZnT8A [23]. In striking contrast, in this present Chinese study, only 67 % of autoimmune diabetes patients were GADA positive, though GADA was again the dominant autoantibody. About 33 % of adult-onset autoimmune diabetes Chinese patients were identified by autoantibodies other than GADA, including IA2A, ZnT8A, and IAA, a much higher frequency than that found in Europe. Though the numbers are small, those Chinese patients with diabetes-associated autoantibodies other than GADA did show a range of clinical differences compared with the type 2 diabetes mellitus cases, which is in line with European adult-onset autoimmune diabetes and consistent with these autoantibodies identifying a cohort of patients clinically different from type 2 diabetes mellitus [21].

Limitations of our study include the possibility that we have detected false positives, despite the high specificity for our autoantibody assays and the repetition of the positive assays to both limit that error and increase the assay specificity. Despite the substantial numbers tested, the low positivity of IA-2A, ZnT8A, and IAA also limits the power of our analysis. Moreover, this present cohort probably excludes patients with the more aggressive form of adult-onset autoimmune diabetes, as we have excluded patients on insulin therapy in order to estimate IAA. In summary, while GADA detects about 90 % of autoimmune adult-onset diabetes patients in Europe, our results indicate that in China screening for diabetes-associated autoantibodies should include autoantibodies other than GADA.

## Appendix

### LADA China investigators and Hospitals

LinongJi, Peking University People’s Hospital (Beijing); XiaohuiGuo, Peking University First Hospital (Beijing); Tianpei Hong, Peking University Third Hospital (Beijing); Jumin Lu, The General Hospital of the People’s Liberation Army (Beijing); ZhangrongXu, The 306th Hospital of the People’s Liberation Army (Beijing); Yingsheng Zhou, Beijing Hospital (Beijing); WeipingJia, Shanghai Jiao Tong University Affiliated 6th People’s Hospital (Shanghai); NingGuang, Shanghai Jiao Tong University Affiliated Rui-Jin Hospital (Shanghai); RenmingHu, Hua Shan Hospital, Fudan University (Shanghai); XinGao, Zhongshan Hospital, Fudan University (Shanghai); Yanbing Li, The First Affiliated Hospital, Sun Yat-sen University (Guangzhou); Huazhang Yang, Guangdong General Hospital (Guangzhou); Shaoda Lin, the First Affiliated Hospital,Shantou University (Shantou); Shenren Chen, the Second Affiliated Hospital, Shantou University (Shantou); Lulu Chen, Union Hospital, Tongji Medical College, Huazhong University of Science and Technology (Wuhan); YanchengXu, Zhongnan Hospital of Wuhan University (Wuhan); Hong Li, First Affiliated Hospital of Medical School of Zhejiang University (Hangzhou); Wei Gu, Second Affiliated Hospital of Medical School of Zhejiang University (Hangzhou); Dawang Wang, The First Affiliated Hospital of Wenzhou Medical School (Wenzhou); Qifu Li, The First Affiliated Hospital, Chongqing Medical University (Chongqin); Gangyi Yang, The Second Affiliated Hospital, Chongqing Medical University (Chongqin); HaomingTian, West China Hospital, Sichuan University (Chengdu); Dalong Zhu, Nanjing Drum Tower Hospital, the Affiliated Hospital of Nanjing University Medical School (Nanjing); Chao Liu, Jiangsu Province Hospital, the First Affiliated Hospital with Nanjing Medical University (Nanjing); QiuheJi, Xijing Hospital, Fourth Military Medical University (Xi’an); Zhongyan Shan, The First Hospital of China Medical University (Shenyang); Jianling Du, First Affiliated Hospital of Dalian Medical University (Dalian); Benli Su, Second Affiliated Hospital of Dalian Medical University (Dalian); Yan Liu, The Norman Bethune 1st hospital of Jilin University (Changchun); HuanqiGe, The Norman Bethune 2nd hospital of Jilin University (Changchun); Yadong Sun, People’s Hospital of Jilin Province (Changchun); Qiang Li, The 2nd Affiliated Hospital of Harbin Medical University (Harbin); Jiajun Zhao, Shandong Provincial Hospital (Jinan); LingliOuyang, The First Affiliated Hospital of Guangxi Medical University (Nanning); YuexinBai, The First Affiliated Hospital of Zhengzhou University (Zhenzhou); YuanmingXue, The First People’s Hospital of Yunnan Province (Kunming); Xulei Tang, The First Affiliated Hospital of Lanzhou University (Lanzhou); Lixin Shi, The Affiliated Hospital of Guiyang Medical College (Guiyang); Xiaoyang Lai, The Second Affiliated Hospital of Nanchang University (Nanchang); Jie Liu, Shanxi Provincial People’s Hospital (Taiyuan); Liyong Yang, The First Affiliated Hospital of Fujian Medical University (Fuzhou); HuijuZhong, Xiangya Hospital of Central South University (Changsha); Zhiguang Zhou, The Second Xiangya Hospital of Central South University (Changsha); Jianying Liu, The First Affiliated Hospital of Nanchang University (Nanchang); Jing Yang, The First Hospital of Shanxi Medical University (Taiyuan); YongdePeng, Shanghai First People’s Hospital (Shanghai).

## References

[1] Zhou Z, Xiang Y, Ji L (2013). Frequency, immunogenetics, and clinical characteristics of latent autoimmune diabetes in China (LADA China study): a nationwide, multicenter, clinic-based cross-sectional study. Diabetes.

[2] Fourlanos S, Dotta F, Greenbaum CJ (2005). Latent autoimmune diabetes in adults (LADA) should be less latent. Diabetologia.

[3] Hawa MI, Kolb H, Schloot N (2012). Adult-onset autoimmune diabetes in Europe is prevalent with a broad clinical phenotype: action LADA 7. Diabetes Care.

[4] Yang L, Zhou ZG, Huang G, Ouyang LL, Li X, Yan X (2005). Six-year follow-up of pancreatic beta cell function in adults with latent autoimmune diabetes. World J Gastroenterol.

[5] Lundgren VM, Isomaa B, Lyssenko V (2010). GAD antibody positivity predicts type 2 diabetes in an adult population. Diabetes.

[6] Lyssenko V, Almgren P, Anevski D (2005). Predictors of and longitudinal changes in insulin sensitivity and secretion preceding onset of type 2 diabetes. Diabetes.

[7] Roh MO, Jung CH, Kim BY, Mok JO, Kim CH (2013). The prevalence and characteristics of latent autoimmune diabetes in adults (LADA) and its relation with chronic complications in a clinical department of a university hospital in Korea. Acta Diabetol.

[8] Liao Y, Xiang Y, Zhou Z (2012). Diagnostic criteria of latent autoimmune diabetes in adults (LADA): a review and reflection. Front Med.

[9] Zhou Z, Ouyang L, Peng J (1999). Diagnostic role of antibodies to glutamic acid decarboxylase in latent autoimmune diabetes mellitus in adults. Chin Med J (Engl).

[10] Dong F, Yang G, Pan HW (2014). The association of PTPN22 rs2476601 polymorphism and CTLA-4 rs231775 polymorphism with LADA risks: a systematic review and meta-analysis. Acta Diabetol.

[11] Lendrum R, Walker G, Cudworth AG, Woodrow JC, Gamble DR (1976). HLA-linked genes and islet-cell antibodies in diabetes mellitus. Br Med J.

[12] Gottlieb DI, Chang YC, Schwob JE (1986). Monoclonal antibodies to glutamic acid decarboxylase. Proc Natl Acad Sci USA.

[13] Li X, Yang L, Zhou Z, Huang G, Yan X (2003). Glutamic acid decarboxylase 65 autoantibody levels discriminate two subtypes of latent autoimmune diabetes in adults. Chin Med J (Engl).

[14] Palmer JP, Asplin CM, Clemons P (1983). Insulin antibodies in insulin-dependent diabetics before insulin treatment. Science.

[15] Huang G, Wang X, Li Z, Li H, Li X, Zhou Z (2010). Insulin autoantibody could help to screen latent autoimmune diabetes in adults in phenotypic type 2 diabetes mellitus in Chinese. Acta Diabetol.

[16] Wenzlau JM, Juhl K, Yu L (2007). The cation efflux transporter ZnT8 (Slc30A8) is a major autoantigen in human type 1 diabetes. Proc Natl Acad Sci USA.

[17] Huang G, Xiang Y, Pan L, Li X, Luo S, Zhou Z (2013). Zinc transporter 8 autoantibody (ZnT8A) could help differentiate latent autoimmune diabetes in adults (LADA) from phenotypic type 2 diabetes mellitus. Diabetes Metab Res Rev.

[18] Yang L, Luo S, Huang G (2010). The diagnostic value of zinc transporter 8 autoantibody (ZnT8A) for type 1 diabetes in Chinese. Diabetes Metab Res Rev.

[19] Xiang Y, Zhou Z, Deng C, Leslie RD (2013). Latent autoimmune diabetes in adults in Asians: similarities and differences between East and West. J Diabetes.

[20] Long AE, Gillespie KM, Rokni S, Bingley PJ, Williams AJ (2012). Rising incidence of type 1 diabetes is associated with altered immunophenotype at diagnosis. Diabetes.

[21] Torn C, Landin-Olsson M, Ostman J (2000). Glutamic acid decarboxylase antibodies (GADA) is the most important factor for prediction of insulin therapy within 3 years in young adult diabetic patients not classified as Type 1 diabetes on clinical grounds. Diabetes Metab Res Rev.

[22] Desai M, Cull CA, Horton VA (2007). GAD autoantibodies and epitope reactivities persist after diagnosis in latent autoimmune diabetes in adults but do not predict disease progression: UKPDS 77. Diabetologia.

[23] Lampasona V, Petrone A, Tiberti C (2010). Zinc transporter 8 antibodies complement GAD and IA-2 antibodies in the identification and characterization of adult-onset autoimmune diabetes: non Insulin Requiring Autoimmune Diabetes (NIRAD) 4. Diabetes Care.

